# Evaluation of Diagnostic Microbiology Capacity and Barriers in Testing for HIV and TB at Peripheral Hospital-Based Laboratories in Oyo-State, Nigeria

**DOI:** 10.1128/spectrum.00459-21

**Published:** 2022-02-09

**Authors:** Oluwayomi T. Bankole, IkeOluwapo O. Ajayi

**Affiliations:** a Department of Epidemiology and Medical Statistics, University of Ibadan, Ibadan, Oyo State, Nigeria; b Department of Biological Sciences, Elizade University, Ilara-Mokin, Ondo State, Nigeria; Memorial Sloan Kettering Cancer Center

**Keywords:** diagnostic microbiology capacity, HIV, TB, peripheral hospital-based laboratory, testing barriers, tuberculosis, human immunodeficiency virus, peripheral hospitals

## Abstract

The prevalence of tuberculosis (TB) and human immunodeficiency virus (HIV) coinfection in Nigeria is currently around 19.1%. This indicates that the two diseases are still a burden on the nation”s health. The aim of this study was to evaluate the diagnostic microbiology capacity and the barriers in performing assay for TB and HIV at peripheral district-level hospital-based laboratories in Oyo State, Nigeria. Diagnostic microbiology capacity was estimated using a scale of 100-point where scores ≤ 49% were categorized as low, 50–79% fair and ≥80% good. Barriers to diagnosis were summarized in proportions. The diagnostic microbiology capacity revealed that 6 (35.3%) and 11 (64.7%) of the laboratories had “fair” and “low” capacity, respectively, to detect TB in cerebrospinal fluid/sputum. In testing for HIV, 3 (17.6%) of the laboratories had “fair capacity” and 14 (82.4%) had “low capacity” to detect CD_4_ count and HIV antibodies in blood serum. The major diagnostic barriers in almost all (94.1%) the laboratories were lack of culture supplies and nonavailability of reagents/testing kits. There was no diagnostic microbiology service with good capacity to facilitate case detection of HIV and TB at the peripheral hospitals. Hence there is a need to improve the supply of reagents, culture stock and testing kits. This will facilitate the detection of TB and HIV cases in peripheral communities.

**IMPORTANCE** This study provided a snapshot knowledge of testing capabilities and commodity availability at state laboratories. The findings should inform the action of stakeholders to improve diagnostic microbiology capacity, consequently enhancing diagnostic measures in detecting human immunodeficiency virus and Mycobacterium tuberculosis.

## INTRODUCTION

Human immunodeficiency virus (HIV) and Mycobacterium tuberculosis are two important infectious agents that still threaten public health in many parts of the world (1). M. tuberculosis causes tuberculosis (TB) and HIV causes acquired immunodeficiency syndrome (AIDS) (2). M. tuberculosis is a non-motile, obligate aerobic, acid-fast, and rod-shaped bacterium (3). HIV is a spherical Lenti-retrovirus, with an envelope that is made up of a lipid bilayer and glycoprotein that enables it to attach to human receptor cells (1). Tuberculosis is mainly transmitted by inhaling aerosols (containing tubercle bacilli) expelled by people who are sick with TB (4). On the other hand, HIV is spread via body fluids such as blood, vaginal secretions, semen, and some other means (5).

Globally in 2015, there were 1.8 million fatalities caused by TB together and at least 400,000 people were living with both HIV and TB (6). In the same year, the range of deaths due to HIV stood around 1.1–1.3 million (7). An estimated 862, 000 people living with HIV worldwide were clinically ill with TB in 2018 (8). Still in year 2018, tuberculosis led the cause of death among people living with HIV (PLWH), which accounted for some 251,000 deaths from HIV-associated TB and about 33% of AIDS deaths (8). The African continent is mostly affected by this important duo, which accounts for 84% of all TB/HIV deaths in 2018 (8). These figures clearly show that HIV and TB remain a great burden to public health in Africa. In Nigeria, about 52,000 deaths were recorded from TB and HIV in 2017 (9). The prevalence of TB and HIV coinfection in Nigeria is currently about 19.1% (10). This suggests that HIV and TB are still a burden on the Nigeria nation’s health. Nigeria is still one of the countries that have the highest burden of TB in the world and highest in Africa (11). In 2017, Oyo State had the 3rd highest burden of TB in Nigeria (12).

Access to and provision of reliable and accurate laboratory diagnosis are essential to prompt detection and control of infectious diseases worldwide (13). However, studies have shown that there are challenges and barriers in testing for diseases especially in rural setting (14), where peripheral hospitals (hospitals located away from major areas of city) are usually located (15). A major challenge in the provision of laboratory services at peripheral hospitals is restricted access to resources for laboratory diagnostic tools and supplies (14). Therefore, improved laboratory diagnostic microbiology capacity at peripheral hospitals is necessary to increase case detection that will reduce the incidence of the HIV and TB when adequate treatment is provided. Hence, this study aimed at evaluating the diagnostic microbiology capacity and the key bottlenecks in testing for TB and HIV at public or government-owned peripheral hospital-based laboratories in Oyo State, Nigeria. These hospital-based or district-level laboratories essentially provide services such as enzyme immunoassays for diagnosis, chemistry, hematology and microbiology, HIV serology, urinalysis, and others.

## RESULTS

### Characteristics of the respondents and laboratory.

Approximately, a response rate of 60% was obtained from the total sampling frame. Other laboratories declined informed consent or refused to participate due to personal reasons. Their right to decline participation was respected. The frequency distribution of the sociodemographics characteristics of the respondents showed that 7 (41.2%) were females and 10 (58.8%) were males. Their ages ranged from 34–51 years with a mean of 42.0 ± 5.1 standard deviation. The mean length of being in service of the respondents at the hospital-based laboratory was 11.9 ± 8.8 years. Thirteen (76.5%) respondents possessed any additional on-the-job professional certification. Nine (53%) of the respondents had a postgraduate education and almost half 8 (47.1%) had first degree called the Medical Laboratory Science degree. Nine (53%) of the respondents had previously undergone on-the-job training on laboratory diagnosis of HIV and TB. All the 17 laboratories were located in either the periphery of the city or rural areas.

### Assay performed at the hospital-based laboratories.

The analysis of assay for TB revealed that 9 (52.9%) of the laboratories could carry out acid-fast bacilli (AFB) smear using Ziehl-Neelsen (ZN) stain and 8 (47.1%) using Rhodamine or Auramine stain. No laboratory could carry out AFB culture and antimicrobial susceptibility test. Out of all the laboratories, 7 (41.2%) could carry out serological test to detect HIV specific antibodies and 3 (17.6%) tests blood for cluster of differentiation 4 (CD_4_) count.

### Diagnostic microbiology capacity for HIV and TB.

Evaluating the overall diagnostic capacity revealed that 6 (35.3%) and 11 (64.7%) of the laboratories had “fair” and “low” capacity, respectively, to test cerebrospinal fluid or sputum or for TB. For HIV, 3 (17.6%) of the laboratories had “fair capacity” and 14 (82.4%) had “low capacity” to test blood serum for HIV antigen’s antibody and blood for CD_4_ count. None of the laboratories had good capacity to test for TB and HIV (Table 1 and 2).

**TABLE 1:** Composite scores of bacteriological examinations of sputum or cerebrospinal fluid for TB test (N = 17)^*a*^

	Staff	SOPs		Equipment		Reagents/test kits		Quality control			External quality assessment			
Study laboratory	Lab. Scientist certified to perform the test	Availability of SOP for test	Up-to-date SOP for test	Availability of equipment for the test	Equipment functioning for the test.	Availability of adequate reagent/kit for test	Reagents in-date	Conduct of IQC during test	IQC result acceptable	Correction done for unacceptable IQC result	Lab participate in EQA for test	EQA result acceptable	Correction done for unacceptable EQA result.	Score per lab
I	100	100	100	100	100	0	0	50	0	0	0	0	0	0.42
II	100	100	100	50	50	0	0	100	100	50	0	0	0	0.50
III	100	100	100	100	100	0	0	0	0	0	0	0	0	0.39
IV	100	100	100	100	100	0	0	100	50	50	50	50	50	0.65
V	100	100	100	50	50	0	0	0	0	0	0	0	0	0.31
VI	100	100	100	100	100	0	0	100	50	50	0	0	0	0.54
VII	100	100	50	0	0	0	0	0	0	0	0	0	0	0.19
VIII	100	0	0	100	100	0	0	0	0	0	0	0	0	0.23
IX	100	0	0	50	50	0	0	0	0	0	0	0	0	0.15
X	100	0	0	50	50	0	0	50	0	0	0	0	0	0.19
XI	100	100	50	0	0	0	0	0	0	0	0	0	0	0.19
XII	100	100	100	100	100	0	0	50	50	50	50	50	50	0.62
XIII	100	100	0	100	100	0	0	0	0	0	0	0	0	0.31
XIV	100	100	100	100	100	100	100	50	0	0	0	0	0	0.58
XV	100	100	100	100	100	0	0	0	0	0	0	0	0	0.38
XVI	100	100	100	100	100	0	0	50	50	50	50	50	50	0.62
XVII	100	0	0	100	100	0	0	50	0	0	0	0	0	0.27

a SOP, standard operating procedure; IQC, internal quality control; EQA, external quality assessment.

**TABLE 2:** Composite scores of serological examinations of serum and blood for HIV tests (N = 17)^*a*^

	Staff	SOPs		Equipment		Reagents/test kits		Quality control			External quality assessment			
Study laboratory	Lab. Scientist certified to perform the test	Availability of SOP for test	Up-to-date SOP for test	Availability of equipment for the test	Equipment functioning for the test.	Availability of adequate reagent/kit for test	Reagents in-date	Conduct of IQC during test	IQC result acceptable	Correction done for unacceptable IQC result	Lab participate in EQA for test	EQA result acceptable	Correction done for unacceptable EQA result.	Score per lab
I	100	100	100	50	50	0	0	0	0	0	0	0	0	0.31
II	100	100	100	100	100	0	0	100	100	50	0	0	0	0.58
III	100	100	100	50	50	0	0	0	0	0	0	0	0	0.31
IV	100	100	100	100	100	0	0	50	50	50	50	50	50	0.62
V	100	100	100	100	100	0	0	0	0	0	0	0	0	0.38
VI	100	100	100	100	100	0	0	50	0	0	0	0	0	0.42
VII	100	100	50	50	50	0	0	0	0	0	0	0	0	0.27
VIII	100	50	50	0	0	0	0	0	0	0	0	0	0	0.15
IX	100	0	0	100	100	0	0	50	50	0	0	0	0	0.31
X	100	0	0	50	50	0	0	0	0	0	0	0	0	0.15
XI	100	100	100	50	50	0	0	0	0	0	0	0	0	0.31
XII	100	100	100	100	100	0	0	100	50	0	0	0	0	0.50
XIII	100	0	0	100	100	0	0	100	50	50	0	0	0	0.38
XIV	100	100	100	100	100	100	100	100	100	50	0	0	0	0.73
XV	100	100	100	100	100	0	0	0	0	0	0	0	0	0.38
XVI	100	100	100	100	100	0	0	100	100	50	0	0	0	0.58
XVII	100	0	0	100	100	0	0	50	50	50	0	0	0	0.35

a SOP, standard operating procedure; IQC, internal quality control; EQA, external quality assessment.

### Barriers in testing for TB and HIV.

At the time of data collection, nearly all the hospital-based laboratories reported that nonavailability of testing kits/reagents - 16 (94.1%), and the lack of culture supplies for acid-fast bacilli - 17 (100%) were the diagnostic barriers for TB and HIV testing. Less than one third of the hospital-based laboratories reported other diagnostic barriers such as absence of laboratory guideline mentioned by 4 (23.5%) respondents and unaffordable test’s cost by 3 (17.6%).

## DISCUSSION

Majority of the peripheral hospital-based laboratories surveyed in this study had “low” diagnostic microbiology capacity in performing assay for TB and HIV, which was attributed mainly to unavailable reagents and/or testing kits and lack of culture supplies specifically for TB. The findings from this study showed that there was no diagnostic microbiology service at the peripheral hospitals with good capacity to facilitate case detection. It also revealed that none of the peripheral hospital-based laboratories functioned in their expected capacity because secondary laboratories are required to perform the assays according to established national guidelines (12). A similar study reported a “drop down” in diagnostic microbiology capacity in district hospital laboratories (16). The “drop down” was attributed to lack of operational requirement such as essential reagents. This observed similarity in both studies was probable because both studies were conducted in resource-poor settings. Other research findings in Africa revealed a poor laboratory capacity in carrying out testing, which was also attributed to lack of laboratory consumables and basic equipment (17).

A recently conducted Nigeria HIV/AIDS Indicator and Impact Survey (NAIIS) revealed that HIV prevalence in Nigeria has reduced from 2.8% to 1.4% and in Oyo State, the figure has declined to less than 1.0% (18). This published decline in prevalence of HIV/AIDS suggests that there have been increased case detection and referrals to clinics for antiretroviral therapy. However, findings from this study contradicted the possibility of increased case detection or testing especially at the peripheral levels because most of the peripheral hospital-based laboratories reported “low” diagnostic capacity for HIV. This could be because the peripheral hospitals are usually marginalized in medical supplies such as laboratory kits/reagents. In a study conducted in Peru, which sought the opinion of medical doctors on diagnostic barriers in rural areas (14), identified deficiencies such as poorly equipped laboratories. Rationally, their finding relates to this study because almost all the hospital-based laboratories reported that unavailable reagents, lack of culture supplies and testing kits were diagnostic barriers.

The reported “low” diagnostic capacity for HIV at the peripheral hospitals could also symbolize a relenting effort in the fight against HIV at the peripheral level. This is dangerous at this time because the battle is not yet won and a resurge in HIV incidence is still possible. Testing is paramount to containing the spread of any infectious disease.

There is a link between rural dwellers, poverty and TB. It has been established that TB is high among rural dwellers (19) and most of the peripheral hospital-based laboratories evaluated in this study were located in the rural areas. Logically, it would be expected that testing services for TB would be high at the peripheral hospitals. The reverse is the case because most of the hospital-based laboratories reported “low” microbiology diagnostic capacity in testing for TB. A diagnostic barrier responsible for this outcome was that the peripheral hospital-based laboratories lacked testing and culture supplies for AFB.

In substantiating the earlier comment on barriers responsible for low testing of HIV, a study has shown that peripheral hospitals in rural settings experience shortage of medical supplies and facilities to test for TB (14). Hence, the observed “low” diagnostic capacity to test for TB could be attributed to shortage of medical supplies. This shortage was supported by majority of the respondents as well. Studies have also shown that the Federal Government has a tendency to prioritize the maintenance of tertiary hospitals at the expense of peripheral hospitals, which are managed by less financially buoyant State Governments (20–22). This practice/approach has led to increased number of health care seekers to laboratories in tertiary institutions that are already congested with demands for testing and care. The approach could also have led to the “low” microbiology diagnostic capacity in testing for TB at the peripheral level. Although, only a few respondents reported nonaffordability of test cost as diagnostic barrier to TB testing, it still validates the findings of Parsons (23) that reported that absence of free or affordable health care services influence perhaps the testing of TB.

In conclusion, the findings from this study showed that there was no diagnostic microbiology service with good capacity to facilitate case detection of HIV and TB at the Oyo State peripheral hospitals. This was due to poorly equipped laboratory with consumables and supplies. The observed “low” capacity of the peripheral hospital-based laboratories in Oyo State calls for interventions to improve the supply reagents, culture supplies and testing kits in order to enhance prompt detection of HIV and TB cases. This recommendation will also ameliorate demands for service and reduce unnecessary rush of health care seekers to laboratories in tertiary institutions that are already congested with demands for testing and care.

## MATERIALS AND METHODS

### Study population and area.

The research was carried out in Oyo State, which is located in Southwest, Nigeria with an approximate land mass of 28,454 kilometers square and an estimated population of about 5,591,598 people (24). The economy of the state is driven majorly through agriculture, which remains the major source of income for a great number of inhabitants (25). The unemployment rate in Nigeria is 21.1%, Southwest Nigeria (15%) and Oyo State (8.8%) and some people engage in petty trading by the road side (26). Nigeria’s gross-domestic-product (GDP) is 235.92 billion dollars, Oyo State’s ($16.1 billion) where over two-thirds of the population work- and live-in rural areas (27). All the levels of health care are well represented in the state with most of the secondary hospitals distributed in semiurban and rural areas. Under the World Health Organization (WHO) tier system, the laboratories studied belong to the district-level of health care delivery, which are expected to offer services such as HIV serology, acid-fast bacilli (AFB), rapid point of care tests, chemistry, bacteriology, microbiology, hematology or blood transfusion services, CD_4_ count, and others (28). According to the WHO tier system and established guidelines by the Medical Laboratory Science Council of Nigeria, these laboratories are expected to be able to perform the basic and essential assays for HIV and TB tests.

### Study size and design.

This study utilized a descriptive cross-sectional design to survey 17 peripheral hospital-based laboratories that consented to participate in the study. The sample size was not estimated because there was limited number (sampling frame was 29) of functioning government-owned district level or secondary hospital-based laboratories in the study area, Oyo State.

### Data collection procedure.

The data were collected from February and May 2014. A standardized WHO laboratory assessment tool (29) was used to collect information on diagnostic microbiology capacity and the key bottlenecks in testing for TB and HIV at peripheral hospital-based laboratories. The tool was adapted and pretested in two laboratories (located in Ibadan, Oyo-State) that shared common characteristics with the study facilities and the final version was obtained. The tool was divided into sections that captured information such as sociodemographics of the participants, diagnostic microbiology capacity for HIV and TB, and barriers to diagnostic testing of the two infectious diseases.

### Data analysis.

The data were validated and checked for completeness. Statistical Package for Social Sciences software version 18 was used for to analyze the data. The diagnostic microbiology capacity of the diseases was evaluated based on competence to carry out quality test, availability of functioning equipment, internal and external quality control and assessment, use of standard operating procedure and availability of potent reagent. A score of 0, 50 and 100 was given to “no”, “partial,” and “yes” responses, respectively, and the mean score was computed (13, 29). “No” represents unexpected response. Using the average score, the diagnostic microbiology capacity was evaluated on a scale of 0–100. Scores ≤ 49% were evaluated “low”, 50–79% “fair,” and ≥ 80% “good” (13, 29). The data were presented in descriptive statistics such as median, mode, mean and standard deviation.

### Ethical considerations.

The study obtained ethical approval from Oyo State Ministry of Health Ethics Committee (Approval code: AD/13/479/569). The Committee is accredited by the National Health Research Committee. A voluntary informed consent duly signed by the participants was also obtained to ensure that the study adhered to ethical principles stated in the Declaration of Helsinki 1964 as revised at the General Assembly in Fortaleza in October 2013. All the information provided by the participants were kept confidential and protected in a locked cabinet. The participants were given a ballpoint pen in appreciation of their participation.
